# Case of functional paraganglioma with intraoperative hypertensive crisis during robot-assisted thoracoscopic resection

**DOI:** 10.1186/s40792-024-01930-w

**Published:** 2024-05-22

**Authors:** Björn Lachmann, Michael Schweigert, Ana Beatriz Almeida, Stephanie Spieth, Thomas Rössel, Torsten Richter

**Affiliations:** 1 Department of Anaesthesiology and Intensive Care Medicine, University Hospital Carl Gustav Carus Dresden, Technische Universität Dresden, Fetscherstr. 74, 01307 Dresden, Germany; 2 Department of Thoracic Surgery, University Hospital Schleswig-Holstein, Campus Lübeck, Ratzeburger Allee 160, 23538 Lübeck, Germany; 3 Department of Radiology, University Hospital Carl Gustav Carus Dresden, Technische Universität Dresden, Fetscherstr. 74, 01307 Dresden, Germany

**Keywords:** Mediastinal paraganglioma, Intrathoracic pheochromocytoma, Surgical treatment, Thoracoscopic resection, Robotic assisted thoracoscopic surgery (RATS)

## Abstract

**Background:**

Mediastinal paragangliomas are rare. Their dangerousness may unfold during surgery, especially if hormonal activity was previously unknown. We report our experience with this case in context to the incidence and localization of atypically located mediastinal paragangliomas in the literature.

**Case presentation:**

A 69-year-old female patient who was scheduled for thoracoscopic resection due to a posterior mediastinal tumor that had been progressing in size for several years and increasing symptoms. The induction of anesthesia, the ventilation of the lungs and the gas exchange after lung separation was uneventful. After initially stable circulatory conditions, there was a sudden increase in blood pressure up to 300/130 mmHg and tachycardia up to 130/min. This hypertensive phase was difficult to influence and required a rapid and consistent use of antihypertensive medication to bring down the blood pressure to reasonable values. The patient stabilized after tumor resection. The postoperative course was unremarkable. During the intraoperative blood pressure crisis, blood was drawn for analysis. These samples showed elevated concentrations of normetanephrine and metanephrine. The tumor subsequently presented as a catecholamine-secreting paraganglioma.

**Conclusion:**

In order to avoid life-threatening blood pressure crises, hormone activity should be ruled out preoperatively in the case of mediastinal tumor, in which a paraganglioma could be considered in the differential diagnosis, especially if there are indications of hypertension in the medical history. Robotic-assisted thoracoscopic resection of the posterior mediastinal tumor was a feasible surgical method, even in the case of unexpected functional paraganglioma.

## Case

**Fig. 1:**
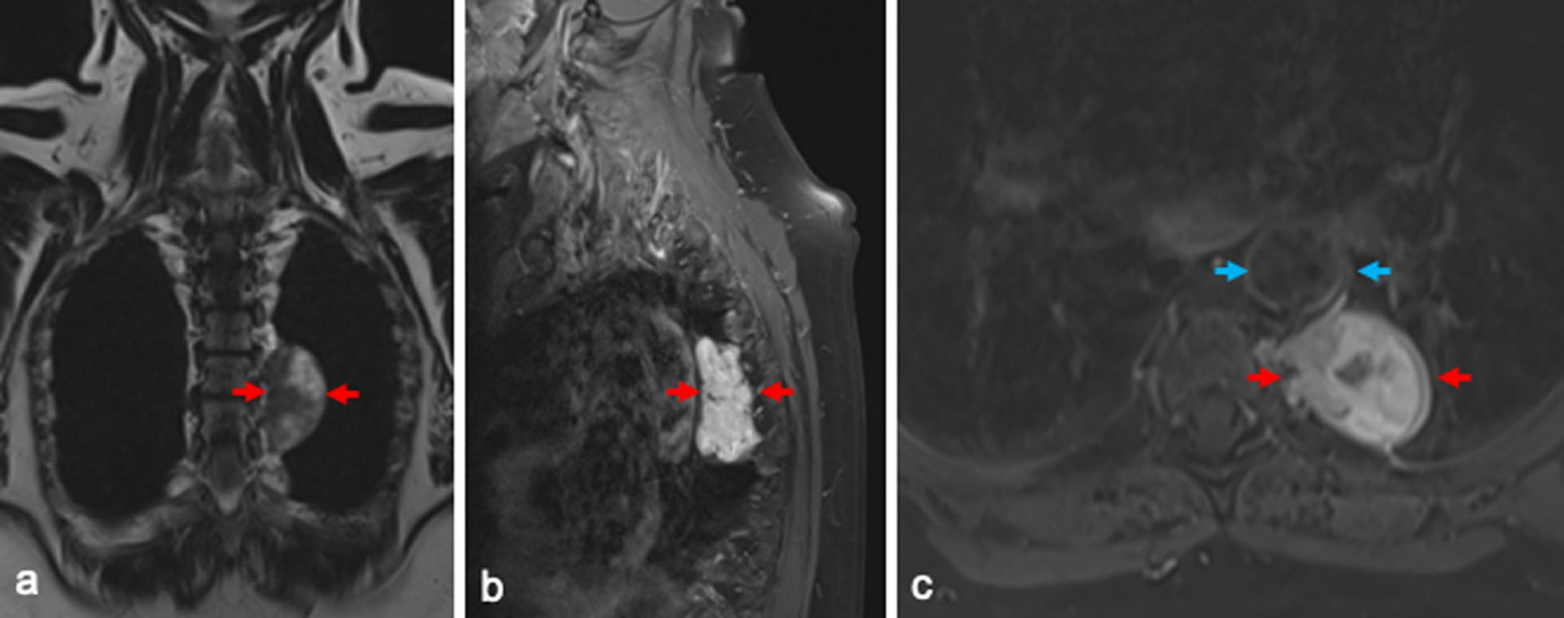
Thoracic magnetic resonance imaging showing the left paravertebral mass with **a** inhomogeneous signal on coronal T2-weigthed images, **b** high signal intensity on sagittal fat-saturated T2-weighted images, and **c** strong inhomogeneous enhancement on axial fat-saturated T1-weighted images. Red arrows show the tumor, adjacent to the aorta marked with blue arrows

A 69-year-old woman had been suffering from an intrathoracic mass with paravertebral localization at the level of the tracheal bifurcation, in the posterior left mediastinum at the level of the 4th to 7th thoracic vertebrae, for 5 years (Fig. 1). With increasing back pain, magnetic resonance imaging (MRI) was performed again. The size of the tumor (37 mm × 39 mm × 64 mm) was found to be progressive and the indication for robot-assisted thoracotomy and tumor removal was given in case of unclear dignity. Retrospectively, a paraganglioma or schwannoma was considered as a differential diagnosis by the radiologists but was not further clarified preoperatively and no serum and urine tests were ordered.

The patient also complained of recurrent pectanginal symptoms and shortness of breath. At least two episodes of hypertensive disorders requiring hospitalization have been described with adjustments to antihypertensive medication. Acute coronary syndrome due to recurrent chest pain was repeatedly ruled out by negative troponin values. No further endocrinological examinations were carried out. A cardiac catheter examination in 2017 revealed a 50% stenosis of the LAD, which was treated conservatively. Echocardiography showed a normal cardiac ejection fraction, mild-left ventricular hypertrophy without relevant lesions. There were no significant changes in the follow-up examinations.

She is self-adjusted with a five-drug combination of antihypertensives (candesartan, lercanidipine, carvedilol, moxonidine, molsidomine). The patient's other cardiovascular risks include obesity (154 cm, 96 kg, BMI: 40.5 kg/m^2^), diet-managed diabetes mellitus, and hyperlipidemia. Hypertensive encephalopathy has also been described.

Furthermore, she is taking atypical neuroleptics for schizophrenia and anxiety disorder and mirtazapine as an antidepressant.

Preoperatively, the patient received a peridural catheter, the placement of which was anatomically difficult (loss of resistance at 10.5 cm). After induction of anesthesia with propofol, sufentanil and rocuronium, intubation with a double lumen tube was performed without any problems. The insertion of a catheter for arterial pressure measurement in the left radial artery and a right internal jugular vein catheter was problem-free. Anesthesia was maintained by continuous administration of propofol (4.0 mg/kg/min), remifentanil (0.5 µg/kg/min), and cis-atracurium (1.0 µg/kg/min) and administration of 0.2% ropivacaine 5 ml/h, continuously + 10 ml ropivacaine 0.3% as an hourly bolus via epidural catheter. The depth of anesthesia was monitored by BIS and ranged from 40 to 50.

Robotic-assisted thoracoscopic surgery (RATS) was performed by using da Vinci Xi^®^ (Intuitive Surgical, Sunnyvale, CA, USA) with 8 mm ports. The positioning of the patient was on her right side. After insertion of the camera trocar in the 8th ICS, the tumor was identified in the mediastinal area, between the spine and aorta, just below the aortic arch. Two further trocars were then placed under direct thoracoscopic view, and the operation was taken over by the console surgeon. After the circular incision of the parietal pleura around the tumor, there was an isolated rise in blood pressure from 130/70 mmHg to 220/100 mmHg, so opioid was administrated additionally, resulting in a brief improvement.

The removal of the tumor from the thoracic wall and from the posterior mediastinum and the free dissection of the tumor was free of technically problems. During this stage of operation, a fulminant blood pressure crisis occurred with an increase to 300/130 (MAP 187) mmHg as well as the increase in heart rate from 80/min to 130/min.

Immediate deepening of anesthesia and administration of a total of 100 mg urapidil and 10 mg metoprolol did not result in sufficient improvement; systolic blood pressure continued to exceed 220 mmHg and was sufficiently lowered by nitroprusside sodium bolus administration followed by continuous i.v. administration via a perfusor. After completely resection of the tumor, there was a significant reduction in blood pressure, so that low-dose catecholamine therapy was now necessary. The patient was ventilated for further monitoring in the intensive care unit and extubated promptly. No further blood pressure spikes occurred. The patient could be discharged from the hospital 5 days after surgery without neurological deficits. The patient recovered without complications.

During the hypertensive crisis, blood was drawn for analysis of catecholamine breakdown products of epinephrine, norepinephrine, and dopamine: metanephrine i.p. 117.8 pg/mL (normal value: < 84.0 pg/mL), normetanephrine i.p. 514.4 pg/mL (normal value: < 190.0 pg/mL), 3-methoxytyramine i.p. 17.2 pg/mL (normal value: < 16.0 pg/mL). Histologic examination of the specimen revealed a picture of paraganglioma without evidence of malignancy.

Postoperatively, all antihypertensive medications were paused, and resumption of medication was not necessary due to the normotensive values. A follow-up endocrinological examination 3 months after surgery revealed values in the normal range for metanephrine, normetanephrine, and 3-methoxytyramine in plasma. Similarly, the patient was referred to the genetic outpatient clinic for further diagnosis.

## Discussion

In the present case, the nature of tumor that was to be operated on was unclear. It was an undiagnosed secretory paraganglioma.

These are rare tumors, and their secretory effects are therefore poorly understood. They originate from sympathetic and parasympathetic cells of the sympathetic trunk and are located in the head and neck, thorax and abdomen. Paragangliomas in the adrenal gland are referred to as pheochromocytomas, which account for approximately 90% of all paragangliomas. They are malignant in up to 35% of cases [1].

Both together have an incidence of about 2–8 cases/1′000′000 people. They are most frequently found in the head and neck region with 69%, 31% are in the thorax and abdomen [2]. In 1–2% of cases they are found in the mediastinum, although only about 150 cases have been described to 2017 [3]. Of all mediastinal tumors, 0.3% are paragangliomas [4].

They are non-secreting in most cases, although this depends on their location. While 4% of paragangliomas are secreting in the head and neck region, about 50% are secreting in the thorax and abdomen. Like pheochromocytoma, they can synthesize the catecholamines epinephrine, norepinephrine, and dopamine, making them a possible cause of secondary arterial hypertension [5, 6]. In up to 50%, they also cause pectanginal and other cardiac symptoms, anxiety, profuse sweating, palpitations, headache, tremor, cough, and shortness of breath. Our patient also exhibited some of these symptoms. Paragangliomas of the posterior mediastinum have been described as non-functional, asymptomatic but functional or usually functional [7].

Paragangliomas are usually removed by surgical resection. If removal is not possible, symptoms are treated with medication. Chemotherapy is only performed in case of malignant findings. However, there are no uniform guidelines on the use of cytostatic drugs for this purpose. Radiation may be considered, but is also performed only in a few cases. Thus, depending on the location, the therapy is very individual.

The surgical resection of thoracic and mediastinal paragangliomas is anatomically challenging. Often, their origin in cells of the truncus sympathicus dictates proximity to major vessels, the heart, trachea, and esophagus. In the reported case, there was a close positional relationship to the adjacent thoracic aorta (Fig. 1).

Variability of mediastinal paragangliomas position determined surgical approach through sternotomy, right or left thoracotomy [8]. In the last decade complete resection by video-assisted thoracoscopic surgery has been reported for anterior [9] and posterior mediastinal paraganglioma [10, 11]. RATS is considered a further improvement for upper thoracic tumor resection as it offers a greater range of motion and is potentially less invasive. Recently, the removal of a rib-invasive posterior mediastinal non-functional paraganglioma combined with en bloc chest wall resection using RATS was assessed [12]. In our case, access and resection of the tumor by RATS was also possible.

The lack of preoperative histological diagnosis, can surprisingly lead to a rapid increase of systolic blood pressure during excision of the pleura around the tumor in a case of an undiagnosed functional paraganglioma [13]. After such life-threatening hypertension was eliminated and the systemic condition was stabilized, we were able to perform RATS entirely, without conversion to a standard left thoracotomy as it was described by using VATS in a similar situation [13].

Paragangliomas often have good vascularization and thus a high risk of bleeding. Therefore, consideration should be given to whether endovascular embolization of the tumor is indicated before surgical resection, as intraoperative tumor bleeding can be severe and sometimes fatal. Biopsy for diagnostic purposes should be avoided if possible in cases of suspected paraganglioma because of the risk of bleeding.

On the other hand, it is important to examine the metabolites of catecholamines in serum and urine, especially in case of corresponding clinical symptoms, which was not done preoperatively in our case. The sensitivity of the metabolites of norepinephrine, normetanephrine as well as dopamine in urine is 90%. MRI and CT have the highest sensitivity in imaging. The primary imaging by SPECT has a sensitivity of 71% because it is poor at detecting smaller tumors. However, it can detect secondary manifestations [2].

If the tumor is a secreting paraganglioma, there is a risk of release of catecholamines during surgical manipulation of the tumor, which in our case led to a hypertensive crisis and tachycardia. This can lead to hypertensive hemorrhage intracerebrally, damage to the myocardium up to infarction or the occurrence of acute pulmonary edema. It is therefore even more important to adjust blood pressure preoperatively [14, 15]. This includes alpha blockade with, for example, phenoxybenzamine or the selective alpha-1 antagonist doxazosin at least 1 to 2 weeks preoperatively. In the case of tachycardia caused by alpha blockade or by the paraganglioma itself, beta blockade can also be performed preoperatively. However, this should only be done after alpha blockade has been performed, as otherwise blood pressure may rise.

Blockade can reduce the incidence of severe blood pressure fluctuations to 4.5% of patients compared to 69% without drug preparation [16]. Several case reports also suggest this reduces perioperative mortality from as high as 45% to 0–3% [14, 15]. As with any surgical procedure, anxiolysis and minimizing perioperative stress is important. Nevertheless, blood pressure fluctuations can occur intraoperatively at any time, as in rare cases there may be clinically and biochemically silent paragangliomas that can still cause blood pressure crises when manipulated. The agent of choice for these hypertensive crises so induced is nitroprusside sodium i.v., which has also been shown to lower blood pressure to an acceptable level in this case. Calcium channel blockers such as clevidipine may also be considered for this use [17, 18]. In this case, an intraoperative event of abnormal intraoperative hypertension could most likely have been avoided if the cause of the hypertension had been more carefully investigated and correctly diagnosed before surgery.

A retrospective study published in 2020 investigated the effects of hypertension and hypotension under paraganglioma and pheochromocytoma resection on postoperative complications [19]. In this study, hypertension and hypotension could be measured for periods of at least 30 min at a systolic blood pressure of > 160 mmHg, at least 10 min at > 180 mmHg, or short-term peak blood pressures of > 200 mmHg, although no maximum time was specified here. No significantly increased rate of postoperative complications was demonstrated in these cases. Nevertheless, blood pressure crises should be avoided and a target pressure of < 160 mmHg should be aimed for over the duration of surgery.

However, it is much more important to avoid intraoperative hypotension because it was associated with an increased rate of acute renal dysfunction, other cardiovascular complications, and prolonged hospitalization. Systolic blood pressure as low as < 95 mmHg for 20 min or systolic blood pressure < 85 mmHg for 10 min and as low as < 80 mmHg by 1 min resulted in significant increases in postoperative complications.

This is not the only reason why postoperative monitoring of patients must always be performed in an intensive care unit or suitable intermediate care unit, as the lack of catecholamine secretion can lead to severe fluctuations in blood pressure, hypotension and hypoglycemia [20].

Patients should continue to be followed and examined during follow-up for several years. Sixty-nine percent are cured by surgical resection, but 31% have recurrence or persistent symptoms from a secondary manifestation of paraganglioma even up to 20 years later [2].

Furthermore, genetic testing should always be arranged, since pheochromocytomas and paragangliomas are hereditary in 25% to 50% of cases. Neurofibromatosis type 1 (Mb. Recklinghausen), von Hippel–Lindau syndrome type 2, multiple endocrine neoplasia (MEN)-type 2, and familial paraganglioma due to mutations in various genes of mitochondrial enzymes are possible genetic causes [21].

## Conclusion

Robot-assisted thoracoscopic surgery was a feasible method for resection of a posterior mediastinal functional paraganglioma. It is important to consider paragangliomas in the case of tumors of the mediastinum, especially in the case of hypertension and in the past medical history. If such a tumor is suspected, the necessary tests should be carried out preoperatively and, in addition to the specific laboratory tests, appropriate medication should be administered if confirmed, as in the case of a pheochromocytoma. In addition, one should be prepared intraoperatively for bleeding complications and severe blood pressure fluctuations, in which hypotension should be avoided. Genetic testing as well as long-term follow-up is necessary for patients at appropriate centers.

## Data Availability

Not applicable. (Laboratory data, MRI images and the protocols associated with the operation are stored in electronic form.)

## Abbreviations

BIS: Bispectral index
BMI: Body mass index
CT: Computed tomography
ICS: Intercostal space
i.pl.: In plasma
i.v.: Intravenously
LAD: Left anterior descending artery
MAP: Mean arterial pressure
Mb: Morbus
MEN: Multiple endocrine neoplasia
min: Minutes
mg: Milligram
mL: Milliliters
mmHg: Millimeters of mercury
MRI: Magnetic resonance imaging
pg: Picogram
RATS: Robotic-assisted thoracoscopic surgery
SPECT: Single-photon emission computed tomography
VATS: Video-assisted thoracoscopic surgery
